# Experiences and challenges of in-hospital rehabilitation exercise among older adults with hip fracture arthroplasty in a fast-track program: a qualitative study

**DOI:** 10.3389/fragi.2026.1761876

**Published:** 2026-05-12

**Authors:** Jianlin Ji, Endong Xie, Yan Li, Hanlin Yang, Ouyao Chen, Lili Chen, Qunfeng Lu

**Affiliations:** 1 School of Nursing, Shanghai Jiao Tong University, Shanghai, China; 2 School of Nursing, The Hong Kong Polytechnic University, Hong Kong, Hong Kong SAR, China; 3 Department of Nursing, Shanghai Sixth People’s Hospital, Shanghai Jiao Tong University School of Medicine, Shanghai, China; 4 School of Nursing, Zunyi Medical University, Zunyi, China

**Keywords:** hip fracture, hip arthroplasty, fast-track recovery, qualitative research, rehabilitation experience

## Abstract

**Introduction:**

Hip fractures are a major global health problem for older adults and fast-track programs are now widely used to improve outcomes. However, older adults following hip fracture arthroplasty often faced difficulties in rehabilitation exercise during hospitalization, and little is known about the factors shaping their exercise behaviors within fast-track care.

**Objective:**

The purpose of this study is to explore the experiences, dilemmas, and needs related to in-hospital rehabilitation exercise among older adults following hip fracture arthroplasty in a fast-track program, to inform strategies for improving rehabilitation support and recovery. Methods: A qualitative interview study was conducted using purposive sampling at a tertiary hospital in Shanghai, China, from January to March 2025. Sixteen older adults with hip fracture arthroplasty aged 65–90 years were recruited. Interviews were audio-recorded, transcribed verbatim, uploaded into NVivo 15.0, and analyzed using thematic analysis.

**Results:**

Four main themes during hospitalization rehabilitation within a fast-track program were identified: (1) Negotiating recovery motivation and rehabilitation-related vulnerability; (2) Disjunction between rehabilitation advice and patients’ understanding; (3) The double-edged role of social networks; (4) Unmet need for rehabilitation support.

**Conclusion:**

This study highlights the urgent need to optimize rehabilitation in older adults after hip fracture arthroplasty, particularly within fast-track care. Optimizing rehabilitation may therefore require greater attention to psychosocial support, clearer and more actionable education, and a more interactive rehabilitation environment, with strengthened involvement from nurses, family caregivers, and peers.

## 1 Introduction

Hip fractures are a global health problem characterized by high morbidity, high disability, and high mortality, and are a heavy burden to healthcare systems worldwide (Ho-Le and Nguyen, 2021; Tian et al., 2025). In 2023, the global number of incident hip fractures among older adults reached over 18,91 million, with China accounting for 19.24% of all cases worldwide (Zhang et al., 2020). As population ageing continues, the burden of geriatric hip fracture is expected to continue to rise (Zhang et al., 2020). As the absolute number of hip fractures in older adults has increased rapidly in China, the total annual hospitalization costs have risen by approximately sixfold (Zhang et al., 2020). The sudden physical trauma from hip fracture not only causes severe psychological distress to the older adults but also significantly reduces their quality of life, leading to restricted mobility and a decline in self-care ability (Taylor et al., 2024). Thus, hip fracture is a major threat to the health and independence of older adults.

Hip arthroplasty is the main treatment for older adults with hip fracture and early, active rehabilitation after surgery is recommended by global guidelines (Min et al., 2021; O’Connor and Switzer, 2022). To promote the rapid rehabilitation, fast-track programs have been widely adopted as a safe and effective model (Sura-amonrattana et al., 2021; Li et al., 2020; Kulshrestha et al., 2019). In fast-track programs, surgery is typically performed within 24–48 h after injury, aiming to shorten hospital stay and improve care efficiency while maintaining quality and safety outcomes. Moreover, shortened hospital stays shift the burden of care to older adults and their families. The skills, knowledge, and confidence developed during hospitalization may also shape their subsequent transition from hospital to home. However, current evaluations of fast-track programs have predominantly focused on clinical and economic metrics (e.g., complications, mortality, costs) (Sura-amonrattana et al., 2021; Li et al., 2020; Kulshrestha et al., 2019; Haugan et al., 2017), while patients’ experiences of initiating and managing rehabilitation exercise have received far less attention.

Rehabilitation exercises are essential for older adults undergoing hip fracture replacement surgery to regain physical function, improve quality of life, and resume independent living. Despite evidence-based recommendations, adherence to rehabilitation exercises remains low during hospitalization. Only 49% of patients engage in rehabilitation exercise on the first postoperative day, and merely 14% maintain daily activity within the first 5 days (Snowdon et al., 2020). Importantly, before hospital discharge, nearly three-quarters of these patients were unable to ambulate independently (Abrahamsen et al., 2022). This substantial gap between guideline recommendations and actual practice highlights the need to understand the dilemmas that shape rehabilitation exercise among older adults with hip fracture arthroplasty during hospitalization.

However, existing qualitative studies on rehabilitation behaviors have largely focused on patients undergoing elective hip arthroplasty for chronic conditions (Chen et al., 2024; Huang et al., 2025). Unlike elective patients who have time to prepare mentally and physically, hip fracture patients suffer from an acute traumatic event, and the adherence of older patients after hip fracture arthroplasty was the lowest (Yan and Chen, 2021). Consequently, their rehabilitation trajectory involves distinct motivational and physical challenges (Marks, 2008). At present, there is limited evidence exists regarding the specific challenges and needs for rehabilitation exercise among older adults with hip fracture arthroplasty within the fast-track program during hospitalization.

Given these gaps, the present study aims to explore the experiences, dilemmas, and needs related to rehabilitation exercises performed during hospitalization in this population within a fast-track program. As the hospitalization period is an initial stage for acquiring rehabilitation skills and building confidence for subsequent recovery, an in-depth understanding of patients’ experiences and requirements during this critical period may help inform strategies to optimize rehabilitation exercise and support more effective recovery under fast-track care.

## 2 Methods

### 2.1 Study design

This qualitative study used semi-structured interviews to explore the rehabilitation experiences and requirements of older adults following hip fracture arthroplasty within the fast-track program. The COREQ checklist is used to report our study (Tong et al., 2007).

### 2.2 Participants and setting

This study was conducted in a tertiary general hospital in Shanghai, China, which implements a fast-track program for older adults with hip fractures. Such individuals are swiftly moved for surgical intervention within 48 h of injury and postoperative hospitalizations stay is usually no longer than 5 days. Bedside rehabilitation is typically initiated by orthopedic nurses and physiotherapists. To ensure sample diversity and representativeness, purposive sampling with maximum variation was employed, capturing a broader range of age, gender, surgical procedure, education level, living conditions, and pre-fracture mobility.

Participant recruitment was initiated by first contacting the head nurses of the relevant departments. The head nurses assisted in identifying potential participants who met the inclusion criteria and informed the search team. However, the head nurses did not determine final participation. Then, the research team approached potential participants in person, provided detailed information about the study, and addressed any questions they had. Those who consented to participate were assured that participation was entirely voluntary, that all personal information would remain confidential, and that they could withdraw from the study at any time without providing a reason or facing any consequences.

### 2.3 Participant eligibility

Participants meeting the following criteria were eligible to participate: (1) patients aged over 65 years; (2) hip fracture patients with hip arthroplasty within the fast-track program; (3) patients had normal walking and weight-bearing ability before injury; and (4) willing to be interviewed and share their experiences.

The exclusion criteria were as follows: (1) patients with other fractures; (2) patients with malignant tumors and blood diseases; (3) patients with a documented history or current psychiatric disorders or cognitive impairment; (4) have complications such as infection or joint dislocation during hospitalization.

### 2.4 Data collection

Face-to-face semi-structured interviews were conducted between January and March 2025. A semi-structured in-depth interview guide was developed to explore participants’ experience, informed by a review of the existing literature on hip replacement recovery. To enhance the guide’s relevance and rigor, consultations were held with a nurse specialist in orthopedic care and a qualitative research expert. The interview guide is provided in Table 1.

**TABLE 1:** Semi-structured interview guide.

Interview guide
1. Could you describe how your hip fracture occurred and what the surgical process was like for you?
2. What do you know about postoperative rehabilitation exercises?
3. What is your attitude toward early ambulation after surgery?
4. Please describe your current situation regarding rehabilitation exercises during this hospital stay
5. What factors facilitate/hinder your rehabilitation exercise?
6. What are your goals for rehabilitation?
7. Do you have any worries or concerns about the rehabilitation process? And what are your expectations or concerns to help you improve your adherence?

The interviews were conducted by the first author and corresponding author in Chinese. Neither interviewer was directly involved in the participants’ routine clinical care at the time of the interviews. Before conducting the interview, participants were informed about the purpose of the study and the focus of the interview. All the participants provided written consent and scheduled an interview time at their convenience. The interview was conducted in a separate private room within the orthopedic ward to ensure privacy and minimize interruptions. Families were not present during the interviews, which helped participants express their experiences more freely. Each interview lasted approximately 30–45 min and was audio-recorded and transcribed verbatim. Patients’ body language, non-verbal expressions, and thoughts and reflections related to the interviews were documented in field notes. The data collection was continued until data saturation was reached, meaning no new information relevant to the research topic emerged.

### 2.5 Data analysis

Transcription of the interviews was completed within 24 h. All audio recordings were transcribed verbatim in Chinese and imported into NVivo version 15 for data management. A bilingual native English speaker, independent of the research team, was hired to translate the transcripts into English, and back-translation was subsequently conducted by another bilingual translator. To preserve semantic and cultural nuances, the original, translated, and back-translated versions were compared by the research team and translators, and any discrepancies or uncertainties were discussed and resolved collaborativelyto ensure accuracy and consistency. All quotations included in this document are translated from the original Chinese interviews.

The transcripts were analyzed systematically using a thematic analysis (Braun and Clarke, 2023). In the first step, two authors independently performed inductive coding. Each author thoroughly familiarized with content by reading the transcripts to gain an in-depth understanding. In the second step, relevant data extracts were systematically coded by annotating notes and codes in the margins of the text. Any disagreements of codes were resolved through third-researcher arbitration and consensus discussion. In the third step, we focused on sorting the different codes into potential themes using NVivo 15.0 software. During the fourth step, the preliminary themes were reviewed, modified, and further developed through recurring team discussions until consensus was achieved. Finally, themes were further refined and defined. The sample size was determined by the principle of data saturation, achieved when three consecutive interviews yielded no new thematic codes and established codes demonstrated repetition. Three additional interviews were conducted following initial saturation to confirm data saturation.

### 2.6 Rigor and reflexivity

The rigor of this study was established according to the criteria of credibility, transferability, dependability, and confirmability. All authors had systematic training in qualitative methods and had prior experience in qualitative research. To ensure credibility, all interviews were conducted by the same researcher using the same semi-structured interview guide developed by comprehensive literature review and consensus by research team. Data saturation was ensured through iterative data collection and analysis. Detailed descriptions of the study context, participant selection criteria, participant characteristics, and procedures for data collection and analysis enhanced the transferability of the results. Dependability was established through an audit trail and peer review of the coding and thematic development. Coding decisions were discussed among the research team to ensure consistency and analytic rigor. Field notes were maintained, and audio recordings and interview transcripts were systematically processed to ensure confirmability. Reflexive practices, including regular team discussions and documentation of potential biases, were employed throughout to enhance the credibility and trustworthiness of the findings.

### 2.7 Ethical considerations

Ethical approval was granted in advance by the Shanghai Sixth People’s Hospital (Ref:2024-205). To ensure anonymity of participants, the digital audio recordings were deleted once the interview data had been transcribed and there was no identifiable information in the transcripts. Participants did not receive financial compensation for taking part in the study.

## 3 Results

We interviewed a total of 16 participants aged between 65 and 90 years, including four males and 12 females. The majority underwent hemi-arthroplasty (n = 11), while five participants received total hip arthroplasty. All fractures were caused by falls. Indicating low-energy trauma as the common injury mechanism. Additional characteristics are available in Table 2. The four main themes and associated sub-themes extracted during the analysis are outlined in Table 3.

**TABLE 2:** Participant characteristics (n = 16).

ID	Age	Gender	Educational level	Surgery	Cause of injury	Living status	POD	Mobility aid
1	84	Female	Junior high school	Hemi-arthroplasty	Fall	Living with spouse	2	No
2	80	Female	Senior high school	Hemi-arthroplasty	Fall	Live alone	4	No
3	71	Female	Junior high school	Hemi-arthroplasty	Fall	Living with spouse	6	No
4	79	Female	Vocational school	Hemi-arthroplasty	Fall	Living with family	9	No
5	76	Male	College	Hemi-arthroplasty	Fall	Living with spouse	1	No
6	79	Male	College	Hemi-arthroplasty	Fall	Living with spouse	2	No
7	79	Male	Junior high school	Hemi-arthroplasty	Fall	Living with family	2	No
8	82	Female	College	Hemi-arthroplasty	Fall	Living with family	2	No
9	74	Female	College	Hemi-arthroplasty	Fall	Living with family	1	No
10	66	Female	Primary school	Total Hip Arthroplasty	Fall	Living with family	3	No
11	90	Female	College	Hemi-arthroplasty	Fall	Live alone	3	No
12	89	Female	Junior high school	Hemi-arthroplasty	Fall	Living with family	6	No
13	77	Female	Senior high school	Total Hip Arthroplasty	Fall	Live alone	1	No
14	69	Female	College	Total Hip Arthroplasty	Fall	Living with family	3	No
15	65	Female	Vocational school	Total Hip Arthroplasty	Fall	Living with spouse	3	No
16	72	Male	College	Total Hip Arthroplasty	Fall	Live alone	2	No

POD: postoperative day.

**TABLE 3:** Four main themes and associated sub-themes.

Themes	Subthemes
Theme 1. Negotiating recovery motivation and rehabilitation-related vulnerability	Internal motivation and goal orientation
	Symptom catastrophizing and self-imposed limitations
	The psychosocial burden of role reversal
Theme 2. Disjunction between rehabilitation advice and patients’ understanding	Unclear dosage, progression, and precautions
	Resigned beliefs and misconceptions about recovery
Theme 3: The double-edged role of social networks	Family, peers, and professionals encouraged rehabilitation
	Overprotection and inadequate caregiving skills limited participation
Theme 4: Unmet needs for rehabilitation support	Need for visualized, quantified, and professionally guidance
	Need for practical training for family caregivers

### 3.1 Negotiating recovery motivation and rehabilitation-related vulnerability

#### 3.1.1 Internal motivation and goal orientation

Many participants expressed a strong desire to recover and reintegrate into a meaningful life, aiming not only to regain basic mobility but also to resume social and recreational activities that symbolized a return to dignity and emotional fulfilment.

I believe I can overcome the challenges of my recovery process and continue living like a normal person, enjoying life with dignity (Participant 9, female, 74).

After some time of rehabilitation, I want to travel with my family, because I really enjoy traveling with them (Participant 12, female,89).

Goal setting also merged as a strategy for sustaining motivation. Some participants actively set specific and measurable goals, ranging from short-term functional improvements—such as incremental increases in exercise volume—to long-term aspirations like the recovery of independent walking ability and resuming normal daily life.

I set myself small goals, like 50 reps today and 100 tomorrow. My biggest wish is to go to the bathroom by myself, that is very important to me (Participant 4, female, 79).

I hope I can recover soon so I can help with cooking and take care of the family again. That’s both my goal and my responsibility (Participant 10, female, 66).

#### 3.1.2 Symptom catastrophizing and self-imposed limitations

Participants’ impression and reinforcement of their physical vulnerability are exaggerated. Rehabilitation exercises can worsen postoperative unpleasant experiences, such pain and bleeding, for certain individuals, which can lead to a vicious cycle that hinders recovery.

My wound was still bleeding, and the drain collected 50 mL. I did not dare move much (Participant 13, female, 77).

I tried to pull up my pants but could not lift my leg; the effort left me sleepless that night due to pain, so I stopped exercising (Participant 16, male, 72).

In addition to objective postoperative complications, self-imposed limitations—such as fear of injury or prosthesis displacement—also emerged as major barriers to early ambulation.

The doctors asked me to get up, but I'm afraid of falling and getting hurt again. I do not feel strong enough. (Participant 13, female, 77).

I have severe osteoporosis, and after such a major surgery, I really do not dare to move around carelessly or get out of bed. If I displace the prosthesis, all of this will be for nothing (Participant 2, female, 80).

#### 3.1.3 The psychosocial burden of role reversal

Most participants had been key caregivers in their families before surgery but were forced into the role of “care recipients” afterwards. This sudden role reversal caused considerable psychological distress, making it difficult for them to focus on their own rehabilitation exercises. Instead, their attention was often redirected toward worrying about family members, which weakened their engagement in recovery.

I used to go to the hospital to accompany my husband and cook for him all the time. Now I’m in hospital, I worry every day about how he can live on his own (Participant 1, female, 84).

I used to be the caregiver at home. My wife has dementia. How is she supposed to live now when I can only lie in bed (Participant 6, male, 79).

In addition, this passive is further reinforced, manifesting as an excessive transfer of rehabilitation responsibility. Some patients completely delegate the responsibility for information gathering and rehabilitation-related decision-making to their family members, demonstrating passive learning and a weakened sense of self-management.

My daughter will help me understand the precautions after this surgery. It does not matter if I know them or not; anyway, my daughter will investigate how I should exercise for me (Participant 12, female, 89).

My daughter-in-law is in charge of my rehabilitation. Every day, she assists me in moving my lower limbs. When she is absent, I do not do any exercise (Participant 10, female, 66).

### 3.2 Disjunction between rehabilitation advice and patients’ understanding

#### 3.2.1 Unclear dosage, progression, and precautions

The main obstacle was not a complete information vacuum, but they emphasized that the guidance they received was often too vague to support safe and confident action. Some participants commonly expressed uncertainty regarding exercise progression, appropriate activity levels, and how to manage potential risks without medical supervision, thereby reducing their confidence and willingness to engage in rehabilitation exercises.

I'm also considering the limits of rehabilitation exercises—how much time and to what extent can I exercise? Everyone tells me to ‘take it gradually’, but nobody explains what ‘gradually’ means or gives any specific numbers. I feel hesitant to exercise (Participant 4, female, 79).

I do not know about some of the contraindications after surgery. If my hip were to dislocate, what symptoms would I have? What should I do? I have no idea. (Participant 5, male, 76).

Rehabilitation guidance often failed to address practical details relevant to daily function as some participants also expressed concerns with the absence of precise instructions for selecting and utilizing rehabilitation equipment.

The doctor said I should not use a low stool, but how low is too low? What kind of chair should I use? There’s no clear standard (Participant 7, male, 79).

The choice of exercise equipment is also quite complicated … I hope there will be clear guidance to help patients choose and use devices correctly to avoid re-injury (Participant 4, female, 79).

#### 3.2.2 Resigned beliefs and misconceptions about recovery

Beyond the lack of specific guidance, some participants viewed recovery as largely beyond their control and to adopt an age-related sense of resignation rather than as a process requiring active participation. Such attitudes were further reinforced by traditional beliefs.

At this age, you cannot force things. If it gets better, great. If not, that’s just life. I also believe it is unnecessary for me to engage in any activities at this moment. After all, as the well-known saying suggests, it takes a hundred days to break the bones (Participant 2, female, 80).

I'm satisfied that the operation is done, and I do not think too much about the future. As long as the operation went well and I can go home, that’s enough. I do not need more than that (Participant 1, female, 84).

Some participants also showed difficulty in seeking, understanding and applying health information, which further deepened their passivity in the rehabilitation.

I do not even know what to ask. How should I ask? Even if I ask, I will not understand the answer (Participant 10, female, 66).

### 3.3 The double-edged role of social networks

#### 3.3.1 Family, peers, and professionals encouraged rehabilitation

Family support played a crucial role in facilitating rehabilitation. Emotional encouragement and consistent companionship made patients feel cared for and empowered.

My daughter shares every little progress I make in our family group. Her encouragement strengthens my motivation (Participant 3, female, 71).

My daughters accompany me, give me encouragement and support, which makes me a lot of confidence (Participant 4, female, 79).

Family also offered hands-on assistance, such as helping with early ambulation, providing essential rehabilitation equipment, and helping patients understand medical information. These actions were seen as tangible expressions of care.

My husband bought me a walking aid and helped me practice. It made me feel like I was truly making progress. (Participant 15, female, 65).

My niece specially helped me to consult and find relevant materials and consulted her classmates in Medical College. Their help made me feel at ease (Participant 7, male, 79).

In addition to family support, peer experiences served as powerful motivators to rehabilitation. Peer examples helped patients overcome fear and uncertainty.

A colleague who had hip arthroplasty after hip fracture told me I must move around to recover. Her encouragement helped me overcame my fear (Participant 3, female, 71).

Last year, my daughter-in-law's father also underwent hip replacement surgery due to a traffic accident. He shared his recovery experience and provided valuable advice that boosted my confidence (Participant 10, female, 66).

Nurses also made an important supportive contribution to rehabilitation. Their encouragement and supervision helped patients feel safer and more confident during ambulation.

During ward rounds, I was encouraged to try getting out of bed. Although I had already tried it before, I was still very afraid to walk. Then, nurses stayed with me and kept telling me that the exercise was safe and helped me when I was scared (Participant 2, female, 80).

At first, I could only stand by the bedside. Later, the nurses kept encouraging and prompting me to do rehabilitation exercises every day, and gradually I was able to walk farther (Participant 3, female, 71).

#### 3.3.2 Overprotection and inadequate caregiving skills limited participation

Some family caregivers lacked professional medical knowledge, had limited time and energy, or experienced strained interactions with patients, which together hindered patients’ engagement in rehabilitation exercises.

My husband wants to help, but he does not know how. He’s afraid I’ll dislocate the joint, so he stops me from doing a lot (Participant 10, female, 66).

My youngest son tells me not to interfere, and he snaps at me whenever I ask anything. Now I just lie here all day, never doing any exercises (Participant 1, female, 84).

### 3.4 Unmet needs for rehabilitation support

#### 3.4.1 Need for visualized, quantifiable and professional support

In response to these uncertainties, participants consistently expressed a strong expectation for professional, visualized and quantifiable rehabilitation support delivered through digital support. Compared with generic online content, they preferred clear and comprehensible instructions for phased rehabilitation.

Some aspects of rehabilitation—such as when to gradually increase intensity or which positions are relatively safe—are still not entirely clear to me. The clinicians in the ward are very busy and cannot go through every detail in depth. I really hope official online platforms or an app with specialized guidance on post-surgery recovery, so that I can follow these digital resources step by step. (Participant 9, female, 74).

I do not believe information from the internet. So, I hope the doctor can provide some written or video instructions through specific digital tools so that I can follow the guidelines during hospitalization to gain knowledge (Participant 7, female, 79).

Some participants also wanted timely feedback during exercise, particularly to increase their sense of safety and reduce uncertainty about whether they were exercising appropriately.

Many patients in the ward, including myself, are a bit afraid of getting injured again during rehabilitation. If there were something that could monitor our condition during exercise and track our daily progress, I think we’d all be more than willing to rehabilitate every day (Participant 9, female, 74).

It would be great if there were a device that could ‘watch over’ me when I'm exercising—like monitoring my heart rate—so I know I'm safe. Without that feedback now, I feel like I'm training blindly in the dark (Participant 16, male, 72).

#### 3.4.2 Need for practical training for family caregivers

Family caregivers were often willing to help but lack competence to provide support. Participants particularly emphasized the need for face-to-face instruction on tasks such as turning over, transferring, and early ambulation to help family caregivers from anxious over-protection to confident, competent support.

I have trouble turning over, and my children hesitate to help because they fear hurting me. We really need someone to demonstrate the harder moves face-to-face and maybe watch us do it once to correct our posture (Participant 7, male, 79).

My daughter is afraid to let me get out of bed because she does not know how to assist me. She only dares to help when the care worker is present. She is terrified that I might fall if she grabs me the wrong way. If someone could provide us with systematic training, we would both feel much more confident (Participant 12, female, 89).

## 4 Discussion

This study deeply explores rehabilitation experiences and needs of older adults with hip fracture arthroplasty within the fast-track program. We identified four main themes: negotiating recovery motivation and rehabilitation-related vulnerability; disjunction between rehabilitation advice and patients’ understanding; the double-edged role of social networks, and unmet needs for rehabilitation support. Overall, our findings underscore a critical gap: while fast-track care is effective in treatment, they may overlook the psychosocial and adaptive support needed to sustain exercise adherence and functional recovery. These insights provide an important direction for improving rehabilitation practices for older adults with hip fracture arthroplasty in fast-track care.

This study showed the complex experiences of older adults with hip fracture arthroplasty. They expressed a desire to recover mobility and resume daily functioning, while their motivation was often constrained by perceived bodily fragility, fear of falling and prosthesis displacement. This aligns with the fear-avoidance model (Alaiti et al., 2025), where catastrophic symptom appraisal leads to movement avoidance and persistent functional restriction. Furthermore, our study found problems related to role adaption that could take focus away from self-rehabilitation and reinforce the passive sick role, transferring rehabilitation responsibility to family members (Sagen et al., 2024; Zhang et al., 2024). These problems we found suggest an insufficient focus on the psychological factors. In the first week following hip arthroplasty surgery, patients are more likely to face psychosocial issues, yet their psychological requirements are frequently disregarded (Chen et al., 2023). Therefore, greater attention should be paid to emotional and psychosocial assessment. Nurses may help patients develop more rational interpretations of symptoms and guide the transformation of negative emotions into more adaptive, positive psychological experiences in future practice.

The information dilemma identified in this study reflected a common challenge among older adults after hip fracture arthroplasty. In this study, we found that participants are constrained by limited understanding, traditional misconceptions about rehabilitation and insufficiently actionable advice. Fan et al. (2023) also found that older adults often struggle to retain large volumes of information and what they do remember is often incomplete or inaccurate. Therefore, addressing this dilemma requires a shift from information dissemination to cognitive empowerment, helping patients to understand, internalize, and apply key messages. Teach-back is recommended as an effective and simple educational approach to improve knowledge, skills and self-care abilities (Talevski et al., 2020). Similarly, Lu et al. (2022) reported that Teach-Back education helped older adults after hip fracture arthroplasty remember rehabilitation information more accurately, while also reducing fear and enhancing self-efficacy.

Importantly, in fast-track care, understanding and implementing rehabilitation is not the responsibility of patients alone. Family members’ knowledge is crucial for patients’ rehabilitation, which is in line with Ma et al. (2026). However, in this study, families played a dual role in rehabilitation. Family members often provided emotional encouragement and practical help, but their support was limited by insufficient rehabilitation knowledge and low caregiving proficiency. Smith et al. (2021) have shown that providing patient-caregiver dyads with information on rehabilitation exercise, practice schedules, and goal setting plans alongside face-to-face skills training not only improves patient health outcomes but also reduces caregiver burden. Based on the present findings, and supported by previous literature, a patient-caregiver dyad approach may be a promising strategy. In future practice, nurses could integrate Teach-Back into routine practice and deliver caregiver–patient dyad–based education tailored to individual needs, thereby transforming information into clear, actionable guidance and actively checking whether patients and caregiver have truly understood it.

Additionally, our findings highlight the importance of how rehabilitation information is presented and delivered. Given the short hospital stay in fast-track care, participants in this study highlighted the need for visualized, quantifiable, and professional rehabilitation support through digital tools. Rehabilitation guidance often provided in the form of brochures without an explanation during the short hospital stay and was difficult for older adults with low-literate to read and follow once they are discharged (Miskiewicz et al., 2024; Ko et al., 2021). Digital tools may provide specific benefits by delivering multimedia-based materials that can be viewed repeatedly, cost-effectively, and may be easier for older adults with lower educational level to understand (Backman et al., 2023). Importantly, participants in this study also emphasized the value of timely feedback during exercise, suggesting that digital support may be particularly valuable when it enhances both progress tracking and reassurance about exercise safety. However, such tools are also limited by literacy, usability, and accessibility challenges among clinicians, patients, and caregivers (Backman et al., 2023). Accordingly, future digital interventions for this population should be co-designed with older adults, caregivers and healthcare professionals, and should undergo feasibility and acceptability testing before wider implementation.

A robust external social support network is crucial for maintaining rehabilitation exercise behavior in older adults with hip fracture arthroplasty. In our study, nurses played a supportive role in rehabilitation by offering encouragement, bedside supervision, and reassurance. However, some healthcare providers were the belief that they need to focus more on acute illness first (Haslam-Larmer et al., 2021). This may unintentionally delay patients’ engagement in rehabilitation. Nurses, as the professionals at primary point of care and who spend the most time with patients, are vital to the success of enhanced recovery (Wainwright et al., 2022) and uniquely positioned to bridge this gap between acute care and rehabilitation. In particular, nurses can promote consistent and safe rehabilitation practices by ensuring effective pain management, providing timely feedback on patients’ activity progress, and coordinating care within the multidisciplinary team (Gray et al., 2023). Peer support provides irreplaceable motivation in promoting rehabilitation. Our study found that encouragement and shared experiences from fellow patients helped reduced fear of falling. This is consistent with previous research showing that social support, especially through peer networks, is linked to better psychological wellbeing and improved adherence, thereby enhancing recovery (Yaacobi et al., 2025). Paired rehabilitation training for patients with similar clinical and demographic characteristics may strengthen exercise adherence based on previous studies (Yaacobi et al., 2025), although logistical challenges may limited its feasibility in routine practice. In this context, nurses may help identify suitable patient pairings, and establish designated time periods to optimize the implementation of paired rehabilitation. Therefore, promoting rehabilitation in future practice may require the development of a more interactive ward environment, in which peer modelling and nursing support work together to support patients’ participation in rehabilitation.

## 5 Limitations

This study was conducted in a single unit, perhaps restricting the applicability of the findings to other units. Despite the interviewers were not clinicians and were not directly involved in participants’ usual nursing care, some participants may have nevertheless viewed them as affiliated with the clinical environment, and socially desirable responses therefore cannot be entirely ruled out. Furthermore, this study primarily examined the inpatient rehabilitation phase immediately following surgery. Future longitudinal qualitative research may be conducted to thoroughly investigate the evolving experiences of patients across various rehabilitation stages, thereby offering a theoretical foundation for developing more phased and ongoing intervention strategies.

## 6 Conclusion

Patient experiences of in-hospital rehabilitation within a fast-track program highlight the need to address psychological distress, informational dilemmas and the complex influence of social networks. Rehabilitation support may therefore require clear and actionable guidance, stronger nurse and caregiver support and promoting peer interaction to foster a positive and interactive rehabilitation environment. Digital support may be explored as a strategy to further improve the continuity of rehabilitation support for older adults with hip fracture arthroplasty in future practice. These findings also provide valuable insights for developing more professional and individualized rehabilitation programs to optimize fast-track care.

## Data Availability

The raw data supporting the conclusions of this article will be made available by the authors, without undue reservation.
